# Seasonal Activity Patterns of Captive Arabian Sand Gazelle (*Gazella marica*, Thomas, 1897) in Qatar

**DOI:** 10.3390/ani15060778

**Published:** 2025-03-09

**Authors:** Nima Mahmoud, Romaan Hayat Khattak, Muhammad Ali Nawaz

**Affiliations:** 1 Environmental Science Program, Department of Biological and Environmental Sciences, College of Arts and Sciences, Doha 2713, Qatar; 2 Salam Veterinary Group, Buraydah 51911, Saudi Arabia

**Keywords:** behavioral ecology, scan sampling, *Gazella marica*, Al Reem Biosphere Reserve, captive–breeding ungulates, desert mammals

## Abstract

The Arabian sand gazelle (*Gazella marica*) is a native and highly adaptable species of the Arabian Peninsula. Due to drastic population declines, the species is listed as globally vulnerable. Very little is known about the behavioral ecology of this species in captivity; therefore, this study was designed to investigate the seasonal variations in the activity patterns of Arabian sand gazelles at Al Reem Biosphere Reserve, Qatar. Data were collected in two phases, i.e., summer (September–October 2021) and winter (December 2021–January 2022), for a total of 16 days. Results revealed that feeding and walking (*p* = 0.001) were the dominant activities in both seasons, yet these were higher in summer compared to winter. Likewise, standing, lying down and other activities (social interactions, defecating, maintenance, sexual behaviors) were also higher in summer compared to winter. All these findings suggest that Arabian sand gazelles are adaptable to harsh environments. However, we strongly recommend a year-round investigation on the impacts of humans, feed types and Arabian Oryx on the behavioral activities of Arabian sand gazelles. In addition, we suggest studying the behavior ecology of the wild scattered populations of Arabian sand gazelles for better management of captive breeding stocks.

## Simple Summary

This study examined how Arabian sand gazelles behave in captivity, focusing on seasonal differences in their activity levels. Arabian sand gazelles were observed at Al Reem Biosphere Reserve in Qatar for 16 days during summer and winter. Results obtained revealed that feeding and walking were the most common activities in both seasons, but these and other behaviors like standing and resting were more frequent in summer. The findings suggest the sand gazelles can adapt to both captive environments and seasonal changes. However, it is strongly recommended to further study the impact of factors like human presence, diet, and interactions with other animals on the gazelles’ behavior, as well as studying wild populations to improve captive breeding programs.

## 1. Introduction

Wild animals exhibit variations in their behavior and activity levels throughout the day, and throughout the year [1,2,3,4]. It is believed that individual fitness and survival are directly impacted by the patterns of temporal activity fluctuations, revealing significant aspects of their ecological niche [5]. In conservation biology, studies on animal behavior are crucial, especially for documenting the activity patterns of wild animals in response to several biotic and abiotic factors [6]. Inhospitable habitats such as sandy deserts, tundra’s and high-altitude mountains have a significant impact on a species’ circadian rhythms, including movements, resting, reproductive cycles, and foraging. These harsh surroundings compel the species to explore flexible and adaptive behaviors which are vital for survival [7]. Therefore, understanding the major variables like temperature that affect these activity patterns can help in finding answers to the questions regarding how organisms are able to survive, adapt, and persist in their surroundings [8,9].

Extremely high temperatures (up to 50.0 °C), intense sun radiation, little precipitation, and limited shade cover make deserts the most austere of all the terrestrial habitats [10]. For successful survival and propagation, animals must meet a variety of basic needs during the day, such as foraging along with additional necessities including seeking shelter, resting and social interactions, etc. [11]. However, the non-burrowing mammals residing in hot arid environments such as deserts are constantly exposed to higher temperatures and face limited availability of vital resources, hindering and affecting their normal activity patterns [12]. These animals maintain their body temperature by utilizing one of three adaptations including evaporative cooling, behavioral adaptations or physiological adaptations. Consequently, abnormal fluctuations in activity patterns may threaten the survival of animals inhabiting hot and dry environments [13,14].

To cope with harsh climatic conditions, desert ungulates have evolved through several behavioral adaptations [15]. The primary strategy of desert antelopes to avoid heat stress is to change their daily activity patterns. Dorcas’s gazelles (*Gazella dorcas,* Linnaeus 1758) in the Sahara, goitered gazelles (*Gazella subgutturosa*, Güldenstaedt 1780) in the arid regions of the northern deserts of Central Asia, and springboks (*Antidorcas marsupialis*, Zimmermann 1780) in the Kalahari Desert all exhibit similar behavior during the hot summer months, with two peaks of activity occurring in the early morning and late afternoon [16,17]. Likewise, other species including springboks (*Antidorcas marsupialis*), Kirk’s dik-diks (*Madoqua kirkii,* Günther 1880), beira (*Dorcatragus megalotis*, Menges, 1894) and gemsboks (*Oryx gazella*, Linnaeus, 1758) switch from performing their activities during daylight to performing them during nighttime in order to counter heat stress [15,18,19,20].

The Arabian Peninsula is the intersection of three biogeographic realms, the Western Palearctic, Afro-tropical, and Indo-Malayan, and is home to a large array of wildlife species including 173 terrestrial mammal species [21]. The fauna of the Arabian Peninsula is a combination of species with affinities to the Horn of Africa, Saharo-Sindian, Iranian-Central Asian, and Mediterranean elements, as well as endemic species from Arabia [22]. Of these species, 11 are ungulates and the Arabian sand gazelle (*Gazella marica*, Thomas 1897) is one of them [21].

The Arabian sand gazelle, known as “Al Reem”, is a relatively smaller antelope species, native to the Middle East, specifically the Arabian and Syrian Deserts. This species was once widely distributed in the aforementioned areas; however, the population has drastically declined due to several factors, with poaching being a prominent cause [23,24,25,26]. The extermination of wild populations of Arabian sand gazelles apparently initiated soon after the Second World War, when the building of highways and the accessibility of four-wheel-drive cars opened up areas that were formerly inaccessible to man [27]. According to the International Union for Conservation of Nature (IUCN) red list, the estimated population size of *G. marica* is 1750–2150 and it has been declared globally vulnerable with decreasing population trends [28].

Several measures are taken to restore the species into their native habitats and to reinforce the existing small, scattered wild populations of Arabian sand gazelles, including captive breeding and re-introduction [27]. The practices mentioned above have mainly focused on population monitoring; yet, there is a dearth of information on the behavior of Arabian sand gazelles bred in captivity. It is believed that animals residing in hot arid environments probably cut back on their activities in higher temperatures [29]. If this holds true, we presume that Arabian sand gazelles will be much more active in winter rather than in summer. Therefore, the current study was designed to investigate the seasonal activity patterns of the Arabian sand gazelle to know how they adjust their daily activities to daily temperatures, to help support the management of this threatened ungulate.

## 2. Materials and Methods

### 2.1. Study Area

**Figure 1:**
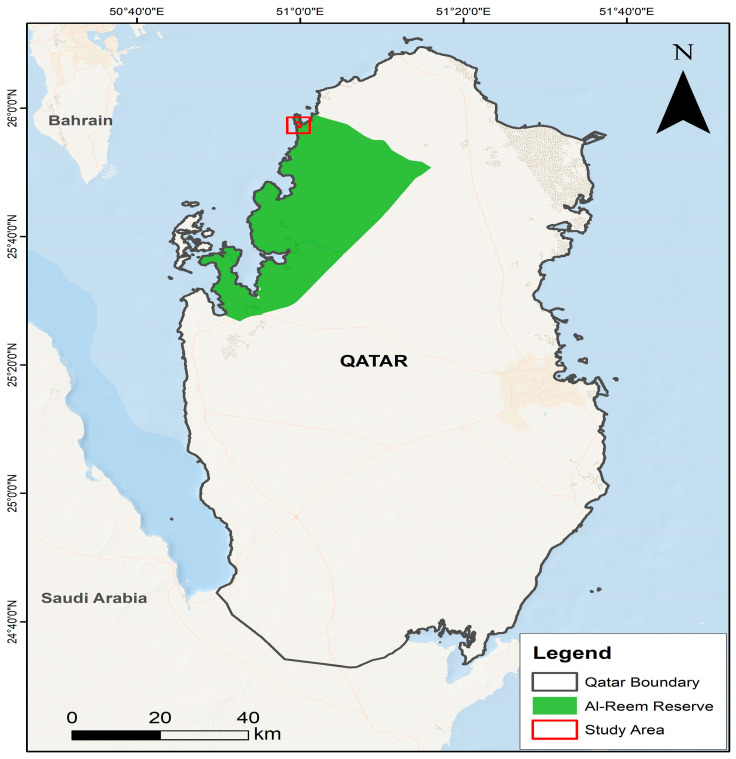
Map showing location of study area.

The current study was carried out at Al Reem Biosphere Reserve (Figure 1). This reserve is located in northwest Qatar (25°53.8′ N, 51°03.00′ E), encompassing an area of 1154 km^2^ with elevation ranges between 0 and 60 m above sea level (asl). It was established as a protected area in 1994 and later on was recognized as a biosphere reserve under UNSECO’s Man and Biosphere (MAB) program in 2007 [30]. The topography of the reserve is characterized by unique gravel plains known as “hazm”, along with saline, swampy, and muddy depressions. Limestone formations are also major topographical features of the reserve. The average annual temperature is 29 °C and this number can hit 40 °C and beyond in summer, with an average annual rainfall of 70 mm. A total of 85 plant species have been reported in the reserve. Key ungulate species of the reserve are the Arabian sand gazelle and Arabian Oryx (*Oryx leucoryx*, Pallas 1777) [31].

### 2.2. Study Animals

Our study animals were located in an outdoor enclosure encompassing an area of 3 × 3 km. This enclosure was a mixed species exhibit, where Arabian sand gazelles (*n* = 360) shared the space with Arabian Oryx (*n* = 400). Our study animals were regularly fed with supplemental feed by the reserve rangers once a day in the morning, including food pellets, fresh feed (green twigs, branches, etc.) and alfalfa hay with an ad libitum supply of water. This reserve also harbored shelters in the form of scattered plantations and manmade shades.

### 2.3. Behavioral Observations

The current study was conducted in two phases, i.e., summer (September–November 2021) and winter (December 2021–January 2022). Data were collected for a total of 16 days (8 days in each season), yielding a sampling effort of 1152 observations in 176 h. Following the methodology of Estes (1991) [18], we designed an ethogram to record activity patterns. We broadly classified the activity patterns in to five categories (Table 1). We employed a group scan sampling method as described by Martin et al. [32]. Based on the time interval method, each observation was recorded after every five minutes from 06:00 am to 12:00 am and from 13:00 pm to 17:00 pm. Observations were recorded by two observers. To reduce the observers’ presence having an impact on the behavior of the animals, we tried to scan the groups from a distance of at least 100 m or more along with using camouflage [33]. Animals and groups were scanned and behaviors were recorded regardless of gender or age in order to remove any bias in choosing animals for any particular observation session. The mean group size of the animals at the reserve was 16 ± 3. Different groups and solitary animals were randomly considered. Air and soil temperatures were recorded hourly using a thermometer, and were assigned to all observations recorded during that hour.

### 2.4. Analytical Approach

To study the effect of season, time, air and soil temperatures on the Arabian sand gazelles’ activities, we used polynomial regression. The initial model contained all of the following factors. To keep only significant factors, we performed stepwise model selection based on the AIC [34].

$$\begin{array}{l} Response\;\sim \;\alpha_{s}+\beta_{1}\left(Season\right)+\beta_{21}\left(Time\right){+\beta }_{22}\;{\left(Time\right)}^{2}{+\beta }_{23}\;{\left(Time\right)}^{3}+\beta_{31}\;\left(Air\;Temperature\right)\\ \;\;\;\;\;\;+\;\beta_{32}\;{\left(Air\;Temperature\right)}^{2}+\beta_{33}\;{\left(Air\;Temperature\right)}^{3}+\beta_{41}\;\left(Soil\;Temperature\right)\\ \;\;\;\;\;\;+\;\beta_{42}\;{\left(Soil\;Temperature\right)}^{2}+\beta_{42}\;{\left(Soil\;Temperature\right)}^{3}+\beta_{1:21}\;Season\;\;\;Time{+\;\beta }_{1:22}\;Season\;\\ \;\;\;\;\;\;*\;{\left(Time\right)}^{2}{\;+\;\beta }_{1:23}\;{Season\;*\;\left(Time\right)}^{3}+\beta_{1:31}\;Season\;*\;Air\;Temperature\\ \;\;\;\;\;\;+\;\beta_{1:32}\;{\left(Season\;*\;Air\;Temperature\right)}^{2}+\beta_{1:33}\;{\left(Season\;*\;Air\;Temperature\right)}^{3}\\ \;\;\;\;\;\;+\;\beta_{1:41}\;\left(Season\;*\;Soil\;Temperature\right)+\beta_{1:42}\;{Season\;*\;\left(Soil\;Temperature\right)}^{2}+\beta_{1:42}\;Season\;*\;\\ \;\;\;\;\;\;{\left(Soil\;Temperature\right)}^{3}+\epsilon_{s}\epsilon_{s} \end{array}$$

where β presents the regression coefficient while the subscript presents the index of respective explanatory variable. (Time)^2^ + (Time)^3^ presents the quadratic and cubic terms of the time factor. The interaction terms (e.g., Season ∗ Air temperature) present the joint effect of these factors. These terms were included to capture potential nonlinear relationships in the data.

In order to differentiate between the variables used in the models, β values were assigned where β_1_ represents season, β_2_ represents time, β_3_ represents air temperature and β_4_ represents soil temperature. Moreover, a second subscript is assigned to β if same variable used in more than one forms, one represents the straight variables used, two superscript shows the squared effect, three subscript represents the cubic effect. 

Each single behavioral category was treated as a response; hence, a separate model was fitted for the behavioral category. For fitting the linear regression, we used function ‘lm’ in program R version 4.1.2 [35].

## 3. Results

**Figure 2:**
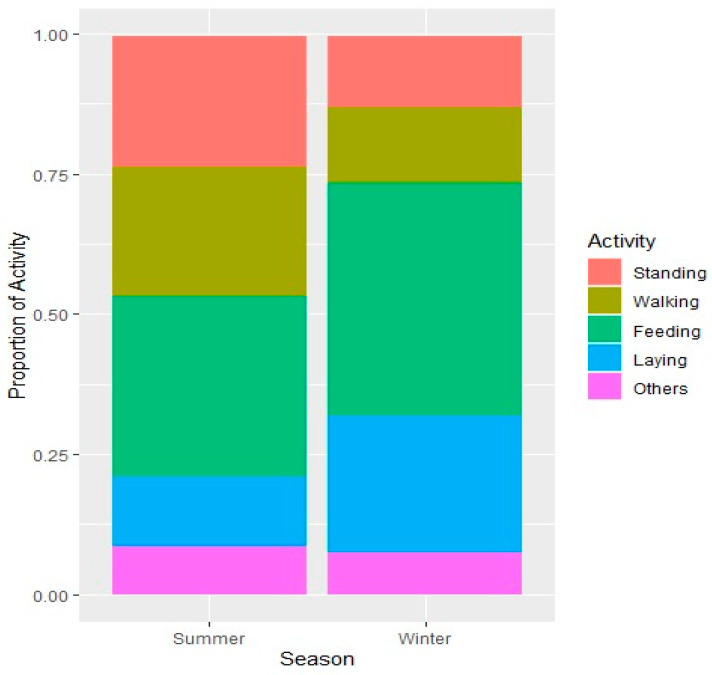
Spread of diurnal time activity budgets of Arabian sand gazelles.

Results obtained in the current study revealed that the season has a profound effect on the time allocation for different activities (Table 2). However, in all categories, the time allocations remained consistent. It is pertinent to note that Arabian sand gazelles achieved this consistency by adjusting the behaviors within each category during the two seasons. For instance, among active behaviors, feeding remained dominant in both seasons, however it was heightened from 32% to 42% in winter at the expense of time allocated for walking. Likewise, in summer, the time allocated for standing was 26% and 13% for lying down, which was reversed to 12% and 24%, respectively, in winter (Figure 2). The final models, selected for each response variable, showed substantial improvements in fit. Corresponding reductions in AIC and improvements in log-likelihood values demonstrated the enhanced predictive power of the final models (Table 3).

**Figure 3:**
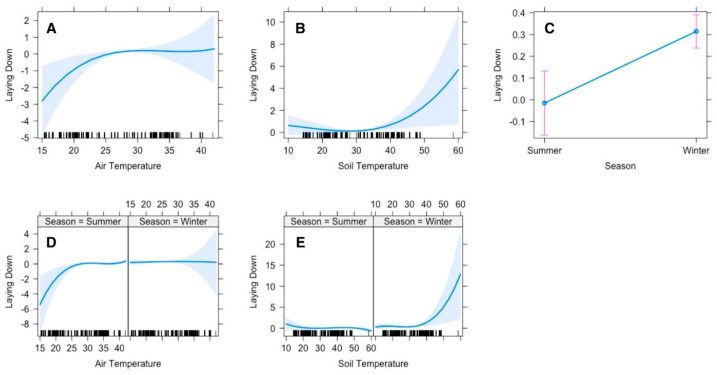
Effect of environmental conditions on a diurnal activity pattern (lying down) of Arabian sand gazelles in Al Reem Reserve, Qatar. The plot illustrates the lying down pattern based on the final selected model. The blue line represents the model’s fitted line, depicting the predicted feeding activity levels throughout the day. The shaded area surrounding the line indicates the 95% confidence interval (CI), providing a measure of uncertainty in the model predictions. The black line on the *x*-axis represents the scale. This visualization offers insights into how environmental factors (**A**–**E**) influence the diurnal feeding behavior of Arabian sand gazelles in Al Reem Reserve.

The top polynomial model suggested that the gazelles’ time allocation for lying down was significantly influenced by season, air and soil temperatures. The gazelles laid down less frequently at lower temperatures (≤15 °C); however, the duration of the lying down increased gradually with increasing temperature until 25 °C, after which there was no noticeable change (Figure 3A). The time allocation for lying down was also influenced by soil temperature. In winter, when the soil temperature was comparatively low, the gazelles laid down less frequently, but when the temperature rose, they tended to lie down more frequently. However, in summer, since the soil temperature was more often high, the animals tended to lie down at any time of the day in shade (Figure 3E).

**Figure 4:**
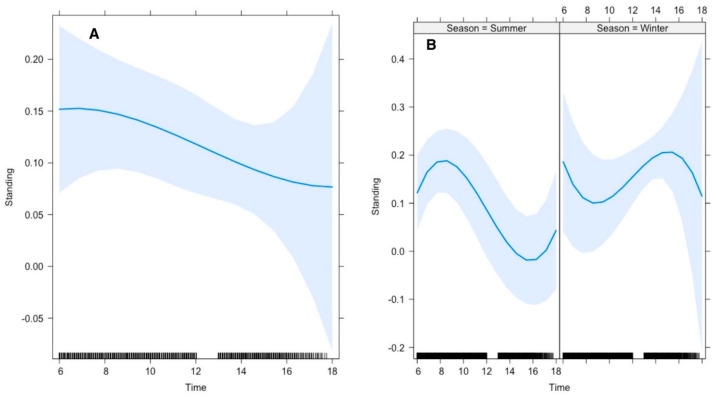
Effect of environmental conditions on a diurnal activity pattern (standing) of Arabian sand gazelles in Al Reem Reserve, Qatar. The plot illustrates the diurnal feeding activity pattern based on the final selected model. The blue line represents the model’s fitted line, depicting the predicted feeding activity levels throughout the day. The shaded area surrounding the line indicates the 95% confidence interval (CI), providing a measure of uncertainty in the model predictions. The black line on the *x*-axis represents the scale. This visualization offers insights into how environmental factors (Time (**A**), and Interaction of Time with Season (**B**) influence the diurnal feeding behavior of Arabian sand gazelles in Al Reem Reserve.

Our results further revealed that the average time gazelles spent standing was 26% in summer and 12% in winter. The top polynomial model suggested that the gazelles’ time allocation for standing was significantly influenced by the time of day. In summer, Arabian gazelles spent significantly higher amounts of time standing during mornings than in afternoons, whereas this pattern was reversed in winter (Figure 4).

**Figure 5:**
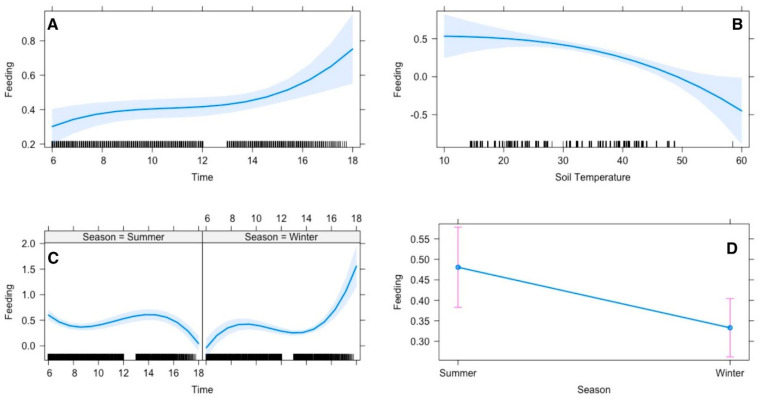
Effect of environmental conditions on a diurnal activity pattern (feeding) of Arabian sand gazelles in Al Reem Reserve, Qatar. The plot illustrates the diurnal feeding activity pattern based on the final selected model. The blue line represents the model’s fitted line, depicting the predicted feeding activity levels throughout the day. The shaded area surrounding the line indicates the 95% confidence interval (CI), providing a measure of uncertainty in the model predictions. The black line on the *x*-axis represents the scale. This visualization offers insights into how environmental factors (**A**–**D**) influence the diurnal feeding behavior of Arabian sand gazelles in Al Reem Reserve.

The average time Arabian gazelles spent feeding was 42% and 32% in summer and winter, respectively. The top polynomial model suggested that the gazelles’ time allocation for feeding was significantly influenced by season, time, and soil temperatures. Feeding activity was lowest in the early morning (30%), there was minor increase until noon, and then a sharp increase was observed in the late afternoon (after 16:00 pm). In the evening (18:00 pm), gazelles utilized the maximum amount of time (80%) feeding (Figure 5A). This pattern is divergent among seasons, as in evenings, feeding activity declined in summer and increased in winter (Figure 5C). If we compare seasons, the Arabian sand gazelles tend to eat more in summer than in winter by 15% (Figure 5D). Moreover, when the soil temperature is low, the gazelles tend to eat more while when the temperature is high, they tend to eat less (Figure 5D).

**Figure 6:**
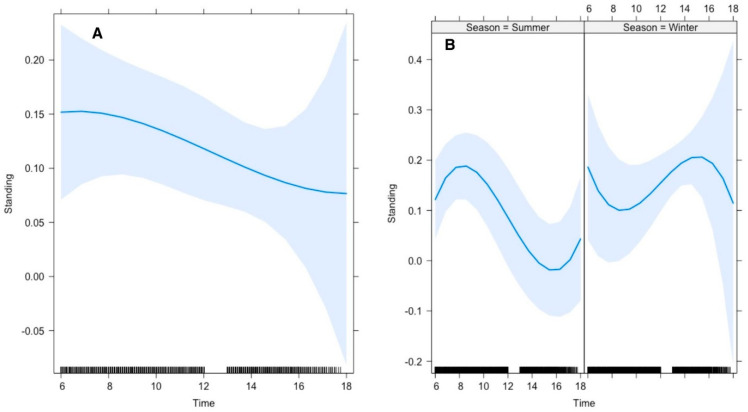
Effect of environmental conditions on a diurnal activity pattern (walking) of Arabian sand gazelles in Al Reem Reserve, Qatar. The plot illustrates the diurnal feeding activity pattern based on the final selected model. The blue line represents the model’s fitted line, depicting the predicted feeding activity levels throughout the day. The shaded area surrounding the line indicates the 95% confidence interval (CI), providing a measure of uncertainty in the model predictions. The black line on the *x*-axis represents the scale. This visualization offers insights into how environmental factors (Time (**A**), and Interaction of Time with Season (**B**) influence the diurnal feeding behavior of Arabian sand gazelles in Al Reem Reserve.

The average time gazelles spent walking was 22% in summer and 14% in winter, respectively. The top polynomial model suggested that the gazelles’ time allocation for walking was significantly influenced by the time of day only. The gazelles began their day by walking then their walking declined dramatically at 12:00 pm, but it started to increase again between 16:00 and 18:00 pm. However, this relationship curve was quadratic (Figure 6). In summer, they walked more in the late afternoon, while in winter, they tended to walk more in the early morning then their amount of time spent walking decreased every hour, until it reached the lowest level in evening (Figure 6).

**Figure 7:**
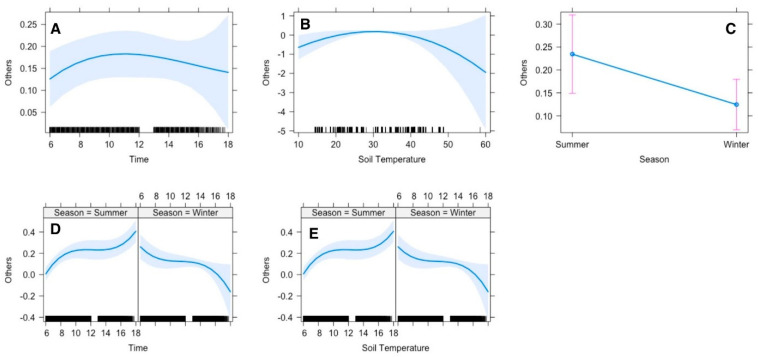
Effect of environmental conditions on a diurnal activity pattern (others) of Arabian sand gazelles in Al Reem Reserve, Qatar. The plot illustrates the diurnal feeding activity pattern based on the final selected model. The blue line represents the model’s fitted line, depicting the predicted feeding activity levels throughout the day. The shaded area surrounding the line indicates the 95% confidence interval (CI), providing a measure of uncertainty in the model predictions. The black line on the *x*-axis represents the scale. This visualization offers insights into how environmental factors (**A**–**E**) influence the diurnal feeding behavior of Arabian sand gazelles in Al Reem Reserve.

Regression analysis suggested that the gazelles’ time allocation for other activities (social interactions, urinating, defecating, grooming, sexual behaviors) was significantly influenced by season and soil temperature. The time gazelles spent in other activities was constant throughout the observation period, which means it could occur at any time of the day (Figure 7A). However, if we compare the two seasons, in summer, they spent much time in other activities in late afternoon, while in winter, the peak time was in the early morning (Figure 7D). In summer, they performed the other activities more than in winter by nearly 25% (Figure 7C). The optimal soil temperature recorded ranged between 25 and 40 °C, within which gazelles tended to perform other activities frequently (Figure 7B). However, in summer, the activities increased every hour until they reached the highest amount of time spent at 18:00 pm, while in winter, the peak time was at 6:00 am with a quadric relationship between them (Figure 7B).

## 4. Discussion

The current study appears to be the first comprehensive attempt to evaluate the seasonal activity patterns of captive Arabian sand gazelles. Results obtained in the current study revealed noticeable impacts of seasons along with other factors like temperature and time of the day on the behavioral displays. Feeding was the most dominant behavioral display among both seasons, yet it was a bit more frequent in the summer compared to the winter. It is reported that desert-dwelling ungulates tend to eat more in summer to meet the energetic costs to cope with heat stress [36]. Our results for the Arabian sand gazelle run parallel to the aforementioned findings. The average annual temperature of the study area was 29 °C, which is high. Keeping in mind the average temperature of the study area, we assume that such a persisting high temperature does not force gazelles to eat much in winter to raise their body temperature.

A negative correlation was found between soil temperatures and feeding, i.e., the lower the soil temperature, the higher feeding rate, and vice versa (Figure 5). Although our study animals were mostly provided with supplemental feed, there can be possible innate behaviors behind this trend. It is reported that gazelles primarily are browsing animals, yet they simultaneously graze and dig soil with their hooves to eat nutritious bulbs and roots [37]. Since the climate of the Arabian Peninsula during the summer is characterized by extreme soil temperatures exceeding up to 60 °C, resulting in no eruption of ground-level forages, this limit feeding [38]. In our finding’s, feeding activity was lowest in the early morning, followed by a minor increase until noon, and then a sharp increase was observed in the late afternoon until evening (after 16:00–18:00 pm). However, our results were slightly contrary to the aforementioned findings for morning hours. We presume that such an observed trend may probably be because of the possible bias in the results due to husbandry and management procedures, the most important of which is the feed supply. Our study animals were sharing the space with the Arabian Oryx which is a dominant and powerful competitor to gazelles. Therefore, we strongly assume that Arabian Oryx thus prevented gazelles from approaching feed once it was served in the morning by rangers, resulting in extended feeding activity by gazelles in the evening. Similar findings have been reported for Indian gazelles (*Gazella bennettii,* Sykes, 1831) living in mixed-species exhibits with Punjab urials (*Ovis vignei punjabiensis,* Lydekker, 1913) [33].

Our results revealed that with a significant impact of time of day only, the time–activity budget for walking by Arabian sand gazelles was 22% in summer and 14% in winter. As it has been mentioned before that summer temperatures in our study area are extremely high [38], we presume that this is a triggering factor of the extensive walking of the Arabian sand gazelles, who were predominantly seeking shelter to avoid heat stress [29]. It is believed that daily excursion activities in wild ungulates reach a peak level at dawn and dusk, with sharp declines in the afternoon [15]. Our findings are supported by the statement mentioned above. However, the compared results for walking in both seasons revealed that Arabian sand gazelles displayed a higher walking frequency at morning in winter, whereas in summer, this trend was observed in late afternoon and evening (Figure 6). We believe that such movement patterns are facilitated by lower temperatures at these hours of the day, thus shifting activities to occur towards cooler periods of the day [15,39].

Lying down and standing were significantly affected by season, air and soil temperatures, and the time of day, respectively (Figure 3). Frequency and duration for lying down were pretty low at ≤15 °C; however, they increased with increasing temperature up to 25 °C. To avoid heat dissipation, it is believed that heterotherms tend to avoid frequent and lengthy body contact with cold soil [40]. Furthermore, gazelles spent more time standing during summer compared to winter. We believe that with rising temperatures (>25 °C), gazelles adopted this strategy to avoid extreme heat stress by lifting their bodies up from ground level [15,39].

In gregarious ungulates, social interactions are of great importance to maintain herd structures and propagation. These interactions included agonistic and affinitive interactions like chasing, blocking, parallel walking, fighting and thrusting, playing, allogrooming, licking and sexual behaviors [33]. Display of the behaviors mentioned above in confined areas is a sign of healthy social enrichment [41]. In addition to staying hygienic, animals also spare time for grooming, namely dust baths which keep the dirt, debris and parasites at bay [42]. Our results revealed that Arabian sand gazelles performed the other activities more in summer than in winter by nearly 25% (Figure 7C), when the optimal temperature recorded ranged between 25 and 40 °C. Defecation was observed much more frequently in summer compared to winter. We attribute this trend to the higher feeding rates displayed by our study animals in summer compared to winter. Although summer temperatures in our study area remain high, having coastal areas in proximity to the study area made the humidity levels considerable [31]. We presume that such a climate is very favorable for the exponential propagation of parasites [43,44]. Therefore, we strongly believe that behaviors like allogrooming, licking and dust baths in Arabian sand gazelles significantly increase in summer compared to winter.

### Study Constraint

Our study has one possible constraint, i.e., that the impacts of Oryx on gazelles and vice versa were not taken in account in terms of interspecific interactions.

## 5. Conclusions

The results obtained in the current study provide insights in into the variations in the seasonal activity patterns of Arabian sand gazelles in captivity. Findings of the current study were counter to our hypothesis, revealing that Arabian sand gazelles are much more active in summer than in winter. In addition, Arabian sand gazelles possess great capability for switching behaviors and trading off, as evident from their walking, lying down and feeding behaviors. Moreover, Arabian sand gazelles displayed higher levels of maintenance behaviors and social interactions in summer than in winter. Since our study animals were also provided with supplemental feed, it is recommended to check the impact of feed types and human presence on the behavior of Arabian sand gazelles. It is advised to investigate the welfare conditions of these animals and to suggest recommendations to ensure optimal animal welfare. It also recommended to perform behavior observations at night using thermal imaging cameras to have detailed information about the 24 h activity patterns of sand gazelles. Furthermore, we strongly recommend long-term monitoring to investigate the effects of the climatic factors and Arabian Oryx on the behavior of the Arabian sand gazelles in the Al Reem Biosphere Reserve, and in the surrounding areas.

## Figures and Tables

**Table 1:** Ethogram designed and used for behavioral observation of Arabian sand gazelles.

S.no	Behavior Pattern	Description of Behavior
1	Walking	When an animal is running or walking from one place to another
2	Feeding	Feeding included foraging, searching for food, ruminating and drinking
3	Lying down	Animal found lying down and still with eyes opened or closed
4	Standing	When an animal is standing still without any signs of alertness
5	Others	Others included social interactions, urinating, defecating, maintenance, and sexual behaviors

**Table 2:** Parameter estimates of polynomial regression models using gazelles’ activities as dependent variables. *p*-values are provided in parenthesis, and parameters with non-significant effects (*p* > 0.05) are excluded. Superscripts “2” and “3” indicate quadratic and cubic effects in the model. NA—Not Applicable.

Variables	Feeding	Walking	Laying Down	Standing	Others
Season (Winter)	−10.3 (1.28 × 10^−8^)	-	27.4 (0.017)	2.46 (0.007)	-
Time	−1.12 1.69 × 10^−21^	-	-	0.38 (0.0058)	-
Time ^2^	0.150 (4.47 × 10^−11^)	−0.03 (0.)	-	−0.034 (0.004)	−0.03 (0.0003)
Time ^3^	−0.003 (3.06 × 10^−11^)	0.001 (0.002)	-	0.0009 (0.006)	0.0009 (0.0008)
Air Temperature	-	-	3.04 (0.005)	-	-
Air Temperature ^2^	-	-	−0.090 (0.005)	-	-
Air Temperature ^3^	-		0.0008 (0.005)	-	-
Soil Temperature	-	-	-	-	−3.04 (0.002)
Soil Temperature ^2^	-	-	-	-	−0.009 (0.0006)
Soil Temperature ^3^	-	-	−7.71 (0.03)	-	8.91 (0.0002)
Season: Winter–Air Temperature	-	-	−3.0 (0.009)	-	NA
Season: Winter–Air Temperature ^2^	-	-	0.091 (0.017)		NA
Season: Winter–Air Temperature ^3^	-	-	−0.0009 (0.039)		NA
Season: Winter–Soil Temperature	-	-	0.62 (0.012)		−0.49 (0.002)
Season: Winter–Soil Temperature ^2^	-	-	−0.02 (0.0071)		0.017 (0.0019)
Season: Winter–Soil Temperature ^3^	-	-	0.0003 (0.0072)		−0.0002 (0.0049)
Season: Winter–Time	3.001 (3.62 × 10^−21^)	-		−0.73 (0.006)	−0.77 (0.00028)
Season: Winter–Time ^2^	−0.27 (7.88 × 10^−21^)	-		−0.06 (0.008)	0.06 (0.0009)
Season: Winter–Time ^3^	0.008 (3.201 × 10^−20^)	-		−0.001 (0.013)	−0.001 (0.0017)

**Table 3:** Comparison of initial and final models for predicting Arabian sand gazelle behaviors based on AIC and log-likelihood values.

Response	Model	AIC	Log-Likelihood
Standing	Initial Model	1089.2	−530.60
	Final Model	970.2	−475.10
Walking	Initial Model	1153.7	−562.85
	Final Model	1005.8	−491.90
Feeding	Initial Model	1102.4	−537.20
	Final Model	987.6	−481.80
Laying	Initial Model	1201.6	−593.80
	Final Model	1018.7	−502.35
Others	Initial Model	1032.5	−504.25
	Final Model	960.1	−460.05

## Data Availability

All the data obtained are presented in this article.
